# Determinants of changes in self-esteem after remission of first-episode psychosis: A study of associated cross-sectional and longitudinal factors

**DOI:** 10.1017/S0033291725102857

**Published:** 2025-12-19

**Authors:** Marit Hidding, Elise van der Stouwe, Bram-Sieben Rosema, Marieke Begemann, Lieuwe de Haan, Jim van Os, Sanne Schuite-Koops, Ben Wijnen, HAMLETT-OPHELIA Consortium, Nynke Boonstra, Wim Veling

**Affiliations:** 1 Psychiatry, UMCG: University Medical Center Groningen, Netherlands; 2 NHL Stenden Hogeschool, Leeuwarden, Netherlands; 3 Biomedical Sciences, UMCG: University Medical Center Groningen, Netherlands; 4 Early Psychosis, Locatie AMC: Amsterdam UMC, Netherlands; 5 Psychiatry, UMC Utrecht: University Medical Center Utrecht, Netherlands; 6 Psychiatry and Neuropsychology, Maastricht University Medical Center, Netherlands; 7 Psychosis Studies, King’s College London, UK; 8 Centre of Economic Evaluation and Machine Learning, Trimbos-Instituut, Netherlands; 9 KieN VIP Mental Health Care Services, Netherlands

**Keywords:** antipsychotic medication, first-episode psychosis, negative side effects, recovery, self-esteem

## Abstract

**Background:**

Low self-esteem is an important and potentially modifiable risk factor for the development and outcome of psychotic disorders. The factors involved in low self-esteem in psychotic disorders are not yet fully understood. The current study aims to investigate the cross-sectional and longitudinal associations between (changes in) self-esteem and severity of psychotic symptoms, internalized stigma, negative reaction to antipsychotics, personal recovery, childhood bullying, childhood trauma, and social support in symptomatically remitted first-episode psychosis (FEP) patients.

**Methods:**

Data from the ongoing longitudinal Handling Antipsychotic Medication: Long-term Evaluation of Targeted Treatment study were used. Participants were in symptomatic remission for 3–6 months after the FEP. Cross-sectional associations (*N* = 299) were investigated through Pearson’s correlations, and longitudinal changes (*N* = 238) were investigated via linear regressions with inverse probability weighting.

**Results:**

Cross-sectionally, we found that lower self-esteem was related to higher severity of symptoms, higher internalized stigma, higher childhood trauma (specifically emotional neglect), higher childhood bullying, more negative side effects of antipsychotic medication, lower personal recovery, and lower social support. Longitudinally, contrary to our hypothesis, we found that higher baseline internalized stigma, higher childhood trauma (specifically emotional abuse), and a higher baseline negative subjective reaction to antipsychotics predicted an increase in self-esteem after 6 months. Furthermore, a decrease in psychotic symptoms, internalized stigma, and negative subjective reaction to antipsychotics, and an increase in social support predicted an increase in self-esteem.

**Conclusions:**

Early intervention programs for psychotic disorders should target factors related to changes in self-esteem. This might improve self-esteem and thereby promote recovery.

## Introduction

Self-esteem can be defined as a subjective evaluation of one’s worth and is an important transdiagnostic psychological factor (Bemrose, Akande, & Cullen, 2020; Donnellan, Trzesniewski, & Robins, 2015; Krauss, Dapp, & Orth, 2023; Struijs et al., 2021). Low self-esteem has been related to the development, severity, and duration of psychotic symptoms and can negatively impact treatment outcomes (Freeman et al., 1998; Krabbendam et al., 2002; MacDougall, Vandermeer, & Norman, 2017; Romm et al., 2011). In first-episode psychosis (FEP) patients, low self-esteem has been associated with distress accompanying the onset of psychotic illness (Vracotas, Schmitz, Joober, & Malla, 2007). Few studies have been conducted to explore the relationship between self-esteem and other factors longitudinally, specifically in people with a psychotic disorder. Given that the early phase of psychosis is often marked by psychological instability, clinical recovery, and exposure to various interventions, self-esteem may be subject to meaningful change during this period (P. Fusar-Poli, McGorry, & Kane, 2017). While cross-sectional studies have provided valuable insights into factors associated with self-esteem in FEP, they do not capture how these associations evolve over time. Furthermore, the factors contributing to low self-esteem in people with psychotic disorders are complex and not yet fully understood (Moritz et al., 2010). A possible explanation is that the view of the self is likely to change during the course of illness, in the process of clinical and personal recovery (Conneely et al., 2021). Moreover, psychotic disorders have been characterized as disorders of the basal self, in which distortions of self-experience are reported (Gram Henriksen, Raballo, & Nordgaard, 2021). These distortions of self-experiences are, for example, feeling alienated from oneself and others and experiencing profound changes in perception of the self and the sense of being in control (Conneely et al., 2021; De Vries et al., 2013). Determinants of self-esteem in individuals with a psychotic disorder might therefore be different than in those with other disorders.

Several studies have found associations between low self-esteem and a range of other factors. The severity of psychotic symptoms was found to be associated with low self-esteem in FEP (Kim et al., 2020; Palmier-Claus, Dunn, Drake, & Lewis, 2011). Internalized stigma has been related to lower self-esteem in people with FEP (Romero-Castillejo et al., 2024). Childhood trauma, including different types of abuse and neglect, as well as other social adversities, have been found to be predictors of low self-esteem in patients with FEP (Morgan et al., 2014). Regarding antipsychotic medications, stigmatizing side effects, such as weight gain, can negatively impact self-image (Conneely et al., 2024; Schimmelmann et al., 2005; Townsend et al., 2022; Yanos, Roe, Markus, & Lysaker, 2008). To our knowledge, no studies on psychotic disorders have investigated the relationship between self-esteem and the discontinuation of antipsychotics; however, fear of relapse has been related to poorer self-esteem (Zukowska et al., 2022). Moreover, previous studies in individuals with FEP have found an association between personal recovery and self-esteem, where lower personal recovery is related to lower self-esteem (Laxmi, Sahoo, Grover, & Nehra, 2023; Maas et al., 2024). Less perceived social support has been linked to lower self-esteem in FEP (Hinojosa-Marqués, Monsonet, Kwapil, & Barrantes-Vidal, 2021). Previous studies have shown that, generally, women tend to have lower self-esteem than men (Bleidorn et al., 2016; Twenge, Carter, & Campbell, 2017). It has also been found that self-esteem changes over time over the life course (Orth, Erol, & Luciano, 2018; Orth & Robins, 2018). Lastly, self-esteem might also be different depending on the Diagnostic and Statistical Manual of Mental Disorders classification, as some classifications (such as schizophrenia) are more stigmatizing than others (Seery, Bramham, & O’Connor, 2021; Vass, Sitko, West, & Bentall, 2017).

Studying changes in self-esteem and associated factors after symptomatic remission from an FEP is important for identifying psychological vulnerabilities that may influence illness progression and functional and personal recovery. Given the dynamic nature of recovery and the potential for psychological change in the months following symptomatic remission, a longitudinal approach is essential to understand which factors contribute to improvements or deteriorations in self-esteem. Gaining insight into these determinants can inform targeted interventions aimed at enhancing resilience, reducing distress, and improving long-term outcomes. Early psychological interventions that address self-esteem may help mitigate the impact of psychosis and support a more positive recovery trajectory (Jordan et al., 2018; Law, Shryane, Bentall, & Morrison, 2016).

The overall aim of the current study was to investigate what factors are most relevant to changes in self-esteem in the year after remission from the FEP. Longitudinal data from the ongoing Handling Antipsychotic Medication: Long-term Evaluation of Targeted Treatment (HAMLETT) study were analyzed (Begemann et al., 2020). The HAMLETT study is a Dutch multicenter, single-blind, randomized controlled trial investigating the effects of dose reduction/discontinuation versus maintenance of antipsychotic medication in people who, at baseline, are in symptomatic remission after FEP for 3–6 months. First, we aimed to investigate the factors related to self-esteem at baseline by examining the associations between self-esteem and severity of psychotic symptoms, internalized stigma, childhood bullying, childhood trauma, negative subjective reaction to antipsychotics, personal recovery, social support, and sociodemographic characteristics (age, sex, education, and diagnosis) cross-sectionally. Then, we aimed to examine whether these factors at baseline predicted changes in self-esteem over a 6-month follow-up period. Finally, we evaluated whether changes between baseline and follow-up in severity of symptoms, internalized stigma, negative subjective reaction to antipsychotics, and social support predicted changes in self-esteem during this period. We hypothesized, based on previous studies, that more severe psychotic symptoms, higher internalized stigma, presence of childhood bullying, higher levels of childhood trauma, a more negative subjective reaction to antipsychotics, lower personal recovery, and lower perceived social support at baseline are associated with lower self-esteem at baseline. Likewise, we hypothesized similar relationships for the associations between these baseline factors and change in self-esteem (i.e. a decrease) between baseline and 6-month follow-up. Lastly, we hypothesized that a decrease in psychotic symptoms, a decrease in internalized stigma, a decrease in negative subjective reaction to antipsychotics, and an increase in perceived social support are associated with an increase in self-esteem.

## Methods

### Data collection and participants

We used data from the ongoing longitudinal HAMLETT study (Begemann et al., 2020). Ethical approval was granted by the Medical Ethics Committee of the University Medical Center in Groningen (UMCG; METC-number 2017–343). All participants provided their written informed consent.

The current study used data from the screening (V1), baseline visit (V2), 3-month post-baseline (V3), and 6-month post-baseline (V4) visits. Data were collected from 2017 to 2023. Participants were between 16 and 60 years old and were in symptomatic remission after FEP for 3–6 months. Full inclusion/exclusion criteria and study procedures can be found in the HAMLETT study protocol (Begemann et al., 2020).

In total, baseline data of 369 participants were available. After the removal of data from participants with incomplete data on the self-esteem measure, data from 299 participants were eligible for cross-sectional analysis. For the longitudinal analysis, baseline and 6 months post-baseline data were available for 292 participants. After the removal of data from participants with incomplete data on the self-esteem measure, data from 238 participants were eligible for the longitudinal analysis.

### Measures

#### Sociodemographic and clinical characteristics

Sociodemographic and clinical characteristics (age, diagnosis, sex, and education) were self-reported and collected during the screening visit using the Comprehensive Assessment of Symptoms and History (Andreasen, Flaum, & Arndt, 1992).

#### Self-esteem

Self-esteem was assessed using the Self-Esteem Rating Scale – Short Form (Lecomte, Corbière, & Laisné, 2006). This self-report questionnaire consists of 20 items that assess positive and negative beliefs about the self and can be answered on a 7-point Likert scale ranging from 1 (*Never*) to 7 (*Always*). Positive and negative beliefs are highly correlated, and a total score was calculated by reversing the negative subscale, so higher total scores reflect higher self-esteem (Vass et al., 2017).

#### Severity of psychotic symptoms

The severity of psychotic symptoms was measured using the Positive and Negative Syndrome Scale (Kay, Fiszbein, & Opler, 1987), a semi-structured interview that evaluates positive, negative, and general psychopathology symptoms over the past 7 days. The items are scored on a Likert scale ranging from 1 (*Not present*) to 7 (*Severe*), with higher scores representing higher symptom severity.

#### Internalized stigma

Internalized stigma was measured using the Internalized Stigma of Mental Illness scale (Hammer & Toland, 2017), a 29-item self-report questionnaire rated on a 4-point Likert scale (1 – *Strongly disagree* to 4 – *Strongly agree*). The items are distributed across 5 subscales: Alienation (6 items), Stereotype endorsement (7 items), Discrimination experience (5 items), Social withdrawal (6 items), and Stigma resistance (5 items). Items in the stigma resistance subscale are reverse-scored before analysis. The total score is calculated by summing all 29 items, with higher scores representing greater levels of internalized stigma.

#### Childhood trauma

Childhood trauma was assessed using the Childhood Trauma Questionnaire – Short Form (Bernstein et al., 2003). This questionnaire consists of 25 items that measure five different types of childhood trauma: physical abuse (5 items), sexual abuse (5 items), emotional abuse (5 items), physical neglect (5 items), and emotional neglect (5 items). The items are answered on a 5-point Likert scale ranging from 1 (*Never true*) to 5 (*Very often true*). A total score for each type of trauma was calculated, as well as a total score, where higher scores represent higher trauma severity.

#### Childhood bullying

Exposure to childhood bullying before the age of 17 years was assessed using the Dutch translation of the short version of the Retrospective Bullying Questionnaire, translated by the European network of national schizophrenia networks studying Gene-Environment Interactions (EUGEI) study (Fusar-Poli et al., 2023; Schäfer et al., 2004). This questionnaire consists of five questions about the presence and severity of the bullying experience. Questions are answered on a Likert scale ranging from 0 (*Never*) to 4 (*Often [weekly]*), and one question about the severity of bullying could be answered on a Likert scale from 0 (*None*) to 3 (*Severe*). A cutoff point of ≥1 for the presence of bullying was used to dichotomize exposure to childhood bullying into 0 = *absent* and ≥1 = *present* for the cross-sectional analysis, conforming to previous studies (Guloksuz et al., 2019).

#### Negative subjective reaction to antipsychotics

Negative side effects to antipsychotics were measured using the Subjective Reaction to Antipsychotics (SRA-34) self-report questionnaire (Lako et al., 2013). It consists of 34 items that are scored on a 3-point scale (No, Yes to a certain degree, Yes to a high degree). The SRA-34 can be divided into undesired (24 items) and desired effects (10 items). The undesired effects consist of nine subscales: Weight and appetite (2 items), Sexual problems (2 items), Slowed down (3 items), Extrapyramidal side effects (2 items), Social withdrawal (2 items), Emotional flattening (1 item), Increased sleep (1 item), Depressive symptoms (1 item), and Other undesired effects (10 items). For the analysis, we utilized only the undesired effects. Higher scores indicate a higher frequency/experience of the undesired effects.

#### Personal recovery

Personal recovery was assessed using the 24-item self-report questionnaire Recovery Assessment Scale (Corrigan et al., 2004; Giffort et al., 1995). Items are scored on a 5-point Likert scale ranging from 1 (*Strongly disagree*) to 5 (*Strongly agree*). Higher scores reflect greater personal recovery. The items are divided into 5 subscales: Personal confidence and hope (9 items), Willingness to ask for help (3 items), Goal and success orientation (5 items), Reliance on others (4 items), and No domination by symptoms (3 items). The three items of the ‘No domination by symptoms’ subscale assumed the presence of mental illness and could be answered as not applicable. Therefore, these items were not added to the total score, and analyses were carried out on the total of the 21 remaining items.

#### Perceived social support

Perceived social support was measured using the Multidimensional Scale of Perceived Social Support (Zimet, Dahlem, Zimet, & Farley, 1988), which consists of 12 items divided over 3 subscales: Significant other (4 items), Family (4 items), and Friends (4 items). Questions are scored on a 7-point Likert scale (1 – *Fully disagree* to 7 – *Fully agree*). A mean score is calculated across all 12 items, representing the total score, where higher scores indicate greater social support.

### Data analysis

The data were analyzed using IBM SPSS version 28. The data analysis consisted of two parts: Cross-sectional and longitudinal analyses.

#### Cross-sectional analyses

Associations between self-esteem and age, sex, education level, and diagnosis were first investigated. For the demographic measures of diagnosis and education level, one-way analyses of variance were used to test whether there was a significant difference in self-esteem scores between the diagnoses and education levels. For the demographic measure of sex, a point-biserial correlation was calculated.

The associations between self-esteem and severity of psychotic symptoms, negative reaction to antipsychotics, personal recovery, childhood bullying, childhood trauma, and perceived social support at baseline were analyzed using Pearson’s partial correlations, controlling for education, sex, age, and diagnosis. All variables were measured at baseline (V2), except for personal recovery, childhood bullying, and childhood trauma; these were only measured at 3 months post-baseline (V3).

#### Longitudinal analyses

To examine whether the baseline levels of severity of psychotic symptoms, internalized stigma, childhood trauma, childhood bullying, negative subjective reaction to antipsychotics, personal recovery, social support, and discontinuation of antipsychotics predicted change in self-esteem over 6 months, and whether change in severity of psychotic symptoms, social support, internalized stigma, and negative subjective reaction to antipsychotics were associated with changes in self-esteem within these 6 months, we conducted a series of separate linear regression analyses. First, weights for each variable were calculated using inverse probability weighting. For each variable, the sample was divided into two groups: a group with a ‘high’ score and a group with a ‘low’ score on the independent variable at baseline using a median split (except for the variables discontinuation of antipsychotics and childhood bullying, which were already dichotomized). Inverse-propensity score weighting was used, where a probability score for each dichotomized variable was calculated using a logistic regression, considering possible confounders (age, sex, diagnosis, and education). Following this, separate linear regression analyses were conducted with self-esteem as the dependent variable. Each independent variable was included as a continuous predictor in its own model, using the assigned inverse probability weights.

## Results

### Cross-sectional analyses

The sample for the cross-sectional analyses consisted of 299 participants, of whom 31.8% were female. The mean age at baseline was 28.3 years (standard deviation [SD] = 8.9; range 17–58, 82.3% 35 years or younger), and 46.7% had pursued higher education. Diagnostic classifications of participants were schizophrenia (*n* = 139), schizophreniform disorder (*n* = 78), schizoaffective disorder (*n* = 69), brief psychotic disorder (*n* = 12), and unspecified schizophrenia spectrum or other psychotic disorder (*n* = 1).

Pearson’s correlations showed correlations between lower self-esteem and higher internalized stigma, lower perceived social support, greater exposure to childhood emotional neglect, higher severity of psychotic symptoms, more negative subjective reaction to antipsychotics, and lower personal recovery. For sex, age, education, and diagnosis, no significant correlations were found with self-esteem. A table with the descriptives and correlations can be found in Table 1.

**Table 1.** Descriptives and partial correlations with self-esteem (SERS-SF) while controlling for sex, age, education, and diagnosis

	Correlations with self-esteem at baseline (SERS-SF)
Severity of symptoms (PANSS)	−.381**
Internalized stigma (ISMI)	−.759**
Childhood trauma (CTQ-SF)	−.146
Emotional neglect	−.206*
Physical neglect	−.027
Emotional abuse	−.113
Physical abuse	−.043
Sexual abuse	−.044
Childhood bullying (RBQ)	−.220*
Negative subjective reaction to antipsychotics (SRA)	−.335**
Personal Recovery (RAS)	.636**
Social Support (MSPSS)	.412**

Abbreviations: CTQ-SF, Childhood Trauma Questionnaire – Short Form; ISMI, Internalized Stigma of Mental Illness; MSPSS, Multidimensional Scale of Perceived Social Support; PANSS, Positive and Negative Syndrome Scale; RAS, Recovery Assessment Scale; RBQ, Retrospective Bullying Questionnaire; SERS-SF, Self-Esteem Rating Scale – Short Form; SRA, Subjective Reaction to Antipsychotics.

* *p* < .05.

** *p* < .001.

### Longitudinal analyses

The sample for the longitudinal analysis consisted of 238 participants, of whom 32.4% were female. The mean age of this sample at baseline was 28.7 years (SD = 9.2; range 17–58, 80.7% 35 years or younger), and 46.2% had pursued higher education. Diagnoses of patients were schizophrenia (*n* = 101), schizophreniform disorder (*n* = 66), schizoaffective disorder (*n* = 60), and brief psychotic disorder (*n* = 11). The mean change in self-esteem was 4.4 (SD = 15.2).

The linear regression analyses showed that higher childhood trauma (specifically emotional abuse), a more negative subjective reaction to antipsychotics, and higher internalized stigma scores at baseline predicted a positive change in self-esteem at 6 months, controlling for possible confounders, including age, sex, education, and diagnosis. Furthermore, a decrease in internalized stigma, an increase in social support, a decrease in severity of symptoms, and a decrease in negative subjective reaction to antipsychotics predicted an increase in self-esteem after 6 months. A full table with means, medians, and results of each linear regression analysis can be found in Table 2.

**Table 2.** Predictors of self-esteem level at 6 months post-baseline

	Mean (SD)	Median (IQR)	*β*	*t*	Sig
Self-esteem					
Baseline	97.5 (20.9)	98.5 (29.0)	N/A	N/A	N/A
6 months post-baseline	101.9 (20.6)	104 (30.3)	N/A	N/A	N/A
Change	4.4 (15.2)	4.0 (16.0)	N/A	N/A	N/A
Severity of symptoms (PANSS)					
Baseline	44.3 (9.8)	42.0 (14.0)	.110	1.707	.089
Change	−1.7 (9.1)	−2.0 (10.3)	−.213	−3.342	<.001
Internalized stigma (ISMI)					
Baseline	55.0 (13.2)	56.0 (20.0)	.283	4.533	<.001
Change	−3.7 (11.6)	−3.0 (11.0)	−.574	−10.758	<.001
Childhood trauma (CTQ-SF)	38.3 (10.7)	36.0 (12.0)	.168	2.529	.012
Emotional neglect	11.1 (4.3)	10.0 (6.0)	.121	1.826	.069
Physical neglect	7.1 (2.4)	6.0 (4.0)	.069	1.043	.298
Emotional abuse	8.2 (3.7)	7.0 (5.0)	.213	3.280	<.001
Physical abuse	5.8 (1.9)	5.0 (1.0)	.029	0.428	.669
Sexual abuse	5.9 (2.7)	5.0 (0.0)	.094	1.422	.156
Childhood bullying (RBQ; present, %)	132^a^ (70.6%)	N/A	−.044	−.551	.582
Negative subjective reaction to antipsychotics (SRA)					
Baseline	12.9 (7.2)	12.0 (11.0)	.249	3.894	<.001
Change	−2.2 (5.5)	−2.0 (7.0)	−.274	−3.234	.002
Personal recovery (RAS)	80.8 (11.3)	81.0 (14.0)	−.068	−1.001	.318
Social support (MSPSS)					
Baseline	5.5 (1.1)	5.75 (1.3)	−.056	−.862	.389
Change	0.06 (0.8)	0.00 (0.9)	.281	4.473	<.001
Discontinuation of antipsychotic medication (yes, %)	153^b^ (64.3%)	N/A	−.048	−.736	.462

Abbreviations: CTQ-SF, Childhood Trauma Questionnaire – Short Form; ISMI, Internalized Stigma of Mental Illness; MSPSS, Multidimensional Scale of Perceived Social Support; PANSS, Positive and Negative Syndrome Scale; RAS, Recovery Assessment Scale; RBQ, Retrospective Bullying Questionnaire; SERS-SF, Self-Esteem Rating Scale – Short Form; SRA, Subjective Reaction to Antipsychotics.

a Of *N* = 157 completed questionnaires at V3.

b With 7 missing of total *N* = 238.

## Discussion

The current study aimed to examine the associations between baseline self-esteem and baseline severity of symptoms, internalized stigma, childhood adversities (trauma and bullying), negative subjective reaction to antipsychotics, personal recovery, and perceived social support. We aimed to identify factors that predict a change in self-esteem after remission from a FEP.

Our results show that lower baseline self-esteem was related to all investigated determinants at baseline, except the sociodemographic variables (age, education, diagnosis, and sex), which is in line with our first hypothesis and previous studies investigating determinants of self-esteem (Kim et al., 2020; Morgan et al., 2014; Palmier-Claus et al., 2011; Romero-Castillejo et al., 2024) Conneely et al., 2024; Schimmelmann et al., 2005; Townsend et al., 2022; Yanos et al., 2008, Laxmi et al., 2023; Maas et al., 2024, Hinojosa-Marqués et al., 2021). However, only higher internalized stigma, higher childhood trauma, and higher negative reaction to antipsychotics at baseline predicted an increase in self-esteem. For childhood trauma, no significant results were found for the specific subtypes of physical abuse, physical neglect, emotional neglect, and sexual abuse. Lastly, we found that a decrease in severity of symptoms, a decrease in internalized stigma, a decrease in negative subjective reaction to antipsychotics, and an increase in social support predicted an increase in self-esteem after 6 months post-baseline.

We found that most of the factors at baseline did not significantly predict a change in self-esteem, which is in contrast with our second hypothesis. For example, experiencing more severe symptoms at baseline did not predict a decrease in self-esteem over time; rather, an increase in severity of symptoms did. This provides evidence for the temporal association between these factors; self-esteem fluctuates together with symptom severity (i.e. a negative relationship), which is in line with previous studies investigating the variability and fluctuations of self-esteem and associations with psychotic experiences (Postma et al., 2021; Thewissen et al., 2007, 2011).

Evaluating the associations with different subtypes of childhood trauma, only higher scores for emotional neglect were significantly correlated to lower baseline self-esteem. Contrary to our hypothesis, overall childhood trauma and specifically higher experienced childhood emotional abuse predicted an increase in self-esteem in 6 months. This finding is difficult to explain. It is most likely a ceiling effect, as those with higher levels of childhood trauma scored lower on both baseline and 6-month post-baseline self-esteem compared to those with lower levels of childhood trauma. Therefore, it is possible that they had more room for improvement. No significant associations were found with the other subtypes of childhood trauma. Other studies have not found such an effect but rather found all subtypes of the Childhood Trauma Questionnaire to be associated with lower self-esteem (Ekinci & Kandemir, 2015; Li & Liang, 2023; Ozakar Akca, Oztas, Karadere, & Yazla Asafov, 2022; Zhou et al., 2024). However, these studies were not conducted in an FEP population. Therefore, these findings might be different for an FEP population. Moreover, overall trauma scores were not that high in the current study, specifically for physical (*M* = 5.8, range 5–25) and sexual abuse (*M* = 5.9, range 5–25), which may explain why we did not observe significant associations between these subtypes and self-esteem.

Another important finding in the current study was that experiencing fewer negative side effects of antipsychotics over time predicted a positive change in self-esteem. However, a higher score at baseline significantly predicted an increase in self-esteem. It could be that in the latter case, there was more room for improvement. Previous studies have described how experiencing (stigmatizing) side effects can negatively impact self-image, stigma, and well-being (Schimmelmann et al., 2005; Townsend et al., 2022; Yanos et al., 2008). This mechanism may explain our findings, but studies on the subjective reaction to antipsychotics and their association with self-esteem are scarce. One study on neuroleptic dosage indeed found that it was significantly negatively associated to self-esteem (Moritz et al., 2010). Also, the highest correlations were found between *D*_2_ receptor occupancy by antipsychotics and items from the Subjective Well-Being Under Neuroleptics Scale that described ‘feeling comfortable, self-confident, and safe’ (De Haan, Lavalaye, Booij, & Linszen, 2005). Our findings highlight the importance of investigating negative side effects and their relation to self-esteem in psychotic disorders. Discontinuation of antipsychotic medication, as studied in the HAMLETT study, did not predict a change in self-esteem. Although some participants maintained their medication dosage, individuals can adapt to medications over time or switch to a different medication, which could have influenced the results (Horowitz et al., 2021). Furthermore, although people maintaining their medication dosage might experience more negative side effects from their medication, which could influence their self-esteem, people who discontinued their medication might have experienced withdrawal symptoms or psychotic symptoms, or had a difficult time adapting to discontinuation of medication, which may have also negatively influenced their self-esteem.

A change in internalized stigma (i.e. decreasing internalized stigma) was found to be a predictor of a change in self-esteem (i.e. increasing self-esteem). This negative relationship between internalized stigma and self-esteem has also been commonly reported in other studies (e.g. Romero-Castillejo et al., 2024). When people with mental disorders, such as schizophrenia, become aware of negative stereotypes associated with the disorder, they can internalize and apply these to themselves (Corrigan, Watson, & Barr, 2006; Horsselenberg, Van Busschbach, Aleman, & Pijnenborg, 2016). These negative thoughts and feelings directly impact self-worth (Corrigan et al., 2006; Corrigan, Larson, & Rüsch, 2009). Higher baseline internalized stigma was a significant predictor of positive change in self-esteem, which again may be attributed to a larger margin for improvement. It is important to note that concepts of self-esteem and internalized stigma overlap, and that there is substantial similarity between the internalized stigma questionnaire and the self-esteem questionnaire.

### Strengths and limitations

The current study used data from the HAMLETT study, which offered a valuable and large dataset on FEP patients in symptomatic remission for 3–6 months. Therefore, many associations with self-esteem could be explored while taking into account possible confounding variables in a relatively homogeneous group of patients. While this may limit generalizability to more stable or chronic populations, it aligns with the study’s aim to identify psychological factors relevant to early recovery. Investigating self-esteem in this context is essential, as this group may be particularly sensitive to psychological changes that influence long-term outcomes. The absence of an assessment of self-esteem before remission limits our ability to assess individual trajectories, and future studies should consider including pre-remission measures to better capture dynamic changes. Another limitation is that participants may have received varying types or intensities of interventions during the 6-month period, which could have influenced changes in self-esteem. These factors were not accounted for in the current analyses. Furthermore, although age was not significantly associated with baseline self-esteem, it was significantly associated with change in self-esteem. Despite being included as a confounder in the weighting procedure, it could still have had direct effects on self-esteem, potentially introducing bias specific to developmental variation. The data used in this study were collected from 2017 to 2023, including when coronavirus disease 2019 restrictions were in place. This could have affected some of the variables in this study, such as social support (due to social distancing). Data on childhood trauma and childhood bullying were collected at a different time point than baseline, namely at 3 months post-baseline. However, because these instruments assess childhood adversities before the age of 17 years, it is not expected that this would influence the results. Furthermore, the amount of missing data varied by instrument, with the most missing data for the childhood bullying questionnaire, due to the later addition of this questionnaire to the study protocol. Additionally, analyses were conducted using only complete cases for the self-esteem measure, which resulted in a reduced sample size. This may have introduced a selection bias if the missing data were not completely at random. For instance, individuals with lower self-esteem or higher psychological distress might have been less likely to complete the entire questionnaire. Excluding these cases could have affected the generalizability of the findings and may have affected the magnitude of the associations. This study also used a global self-esteem score. While this facilitates interpretation, it may obscure potential differences between positive and negative self-esteem, which some studies suggest may change differently in psychosis (Barrowclough et al., 2003; Lecomte et al., 2006). Conversely, other studies argue that these dimensions do not represent distinct constructs, but rather reflect item wording (McKay, Boduszek, & Harvey, 2014; Michaelides, Koutsogiorgi, & Panayiotou, 2016; Monteiro et al., 2022; Urbán, Szigeti, Kökönyei, & Demetrovics, 2014). Lastly, although our study identifies statistically significant associations and predictors, it does not allow for causal inference. Therefore, these associations should be interpreted with caution.

## Conclusion

The current study highlights that self-esteem is an essential component to consider in psychotic disorders, showing many cross-sectional and longitudinal relations to clinically relevant factors (such as severity of psychotic symptoms, negative subjective reaction to antipsychotics, subjective recovery of psychosis, internalized stigma from psychosis, perceived social support, and childhood adversities). This further emphasizes the importance of a holistic treatment approach (National Institute for Health and Care Excellence, 2015). Furthermore, self-esteem itself is an important target for treatment, whereby enhancing self-esteem may, in turn, improve other factors as well – such as personal recovery (Laithwaite et al., 2007; van der Stouwe, Geraets, Rutgers, & Veling, 2021; Zeigler-Hill, 2011). Self-esteem interventions, such as cognitive behavioral therapy have generally been found to be effective for psychotic disorders, although the evidence is somewhat varying (Hall & Tarrier, 2003; Lecomte, Leclerc, & Wykes, 2018; Sönmez et al., 2020). Subjective negative reaction to antipsychotics was found to be a significant predictor of change in self-esteem, which is underrepresented in the literature. This should, therefore, be carefully considered in treatment plans, with a focus on managing adverse effects and adverse attitudes (Stahl, Sy, & Maguire, 2021; Stroup & Gray, 2018). It is important to find the optimal antipsychotic medication, since there are indications that a relatively small subgroup of patients has a substantial negative subjective experience with specific antipsychotics (De Haan et al., 2023). Furthermore, increasing social support, decreasing the severity of symptoms, and reducing internalized stigma appear to be important factors in enhancing self-esteem. However, more longitudinal research is needed to gain a deeper understanding of the complexity of these relationships and potential causal relationships, as well as studies employing the experience sampling method that could further investigate the dynamics of these relationships. Given the paramount importance of self-esteem, future research could explore possible differences in these relationships between FEP and persons in other stages of psychotic disorders.

## Supporting information

Hidding et al. supplementary material Hidding et al. supplementary material

## Data Availability

The data that support the findings of this study are available from the corresponding author upon reasonable request.

## Supplementary material

The supplementary material for this article can be found at http://doi.org/10.1017/S0033291725102857.
