# Supplementary material for: Determinants of changes in self-esteem after remission of first-episode psychosis: A study of associated cross-sectional and longitudinal factors
Source: Psychol Med. 2025 Dec 19;55:e385. doi: 10.1017/S0033291725102857 (PMC13058613; doi:10.1017/S0033291725102857)
Supplement: Hidding et al. supplementary material [file S0033291725102857sup001.docx]

**Appendix A. HAMLETT-OPHELIA Consortium**

**Authors**

Prof. Dr. Iris Sommer1

Prof. Dr. Lieuwe de Haan2

Prof. Dr. Wim Veling3

Prof. Dr. Jim van Os4,5,6

Prof. Dr. Filip Smit7,8,9
Dr. Marieke Begemann1

Dr. Sanne Koops1

Prof. Dr. Machteld Marcelis5,10

Dr. Martijn Kikkert11
Prof. Dr. Nico van Beveren12,13

Prof. Dr. Nynke Boonstra4,14,15

Bram-Sieben Rosema^14^

Dr. P. Roberto Bakker3,11

Dr. Sinan Gülöksüz5,16

Dr. Joran Lokkerbol9

Dr. Ben Wijnen 9

Dr. Bodyl Brand38

Dr. Shiral Gangadin1,41

Erna van ’t Hag3

Dr. Priscilla Oomen37

Dr. Alban Voppel1,17

Franciska de Beer1

Sterre Kamphuis5

Iris Hamers1
Matej Djordjevic3

Toon Scheurink1

Jort Noorman^1^

Prof. Dr. Therese van Amelsvoort5,18

Dr. Maarten Bak5,18
Dr. Steven Berendsen 2,19
Truus van den Brink20

Dr. Gunnar Faber21
Prof. Dr. Koen Grootens22,23

Martin de Jonge 24

Dr. Henderikus Knegtering3,25
Dr. Jörg Kurkamp26
Prof. Dr. Gerdina Hendrika Maria Pijnenborg27,28

Dr. Anton Staring29,36
Dr. Natalie Veen30
Dr. Selene Veerman31
Sybren Wiersma32
Dr. Albert Batalla4
Ruben Curfs33
Jan-Jaap Hage34

Ellen Graveland21
Joelle Hoornaar12
Inge Hobus1,35

Dr. Karin Huizer^33, 39, 40^

**Affiliations**

1 Department of Biomedical Sciences, University of Groningen, University Medical Center Groningen, Groningen, The Netherlands.
2 Department of Early Psychosis, Amsterdam UMC, Academic Medical Center, Amsterdam, The Netherlands.

3 Department of Psychiatry, University of Groningen, University Medical Center Groningen, Groningen, The Netherlands.

4 Department of Psychiatry, UMC Utrecht Brain Center, University Medical Center Utrecht, Utrecht, The Netherlands.
5 Department of Psychiatry and Neuropsychology, School for Mental Health and Neuroscience (MHeNs), Maastricht University Medical Centre, Maastricht, The Netherlands.
6 King's College London, King's Health Partners Department of Psychosis Studies; Institute of Psychiatry, Psychology & Neuroscience, London, United Kingdom.
7 Department of Epidemiology and Biostatistics, Amsterdam Public Health Research Institute, Amsterdam University Medical Centers, location VUmc, Amsterdam, The Netherlands.
8 Department of Clinical, Neuro and Developmental Psychology, Amsterdam Public Health Research Institute, Vrije Universiteit, Amsterdam, The Netherlands.
9 Centre of Economic Evaluation & Machine Learning, Trimbos Institute (Netherlands Institute of Mental Health), Utrecht, The Netherlands.
10 Institute for Mental Health Care Eindhoven (GGzE), Eindhoven, The Netherlands.
11 Department of Research, Arkin Mental Health Care, Amsterdam, The Netherlands.
12 Antes Center for Mental Health Care, Rotterdam, The Netherlands.
13 Department of Neuroscience, Erasmus MC, Rotterdam, The Netherlands.
14 NHL Stenden, University of Applied Sciences, Leeuwarden, The Netherlands.
15 KieN VIP Mental Health Care Services, Leeuwarden, The Netherlands.
16 Department of Psychiatry, Yale University School of Medicine, New Haven, Connecticut.

17 Douglas Mental Health University Center, McGill University, Montréal, Canada.
18 Mondriaan Mental Health Care, Heerlen, The Netherlands.
19 Dimence Institute for Mental Health, Deventer, Zwolle, The Netherlands.
20 Early Intervention Team, GGZ Centraal, Amersfoort, The Netherlands.
21 Yulius, Mental Health Institute, Dordrecht, The Netherlands.
22 Reinier van Arkel Institute for Mental Health Care, 's Hertogenbosch, The Netherlands.
23 Tranzo, TSB, Tilburg University, Tilburg, The Netherlands.

24 Program for Psychosis & Severe Mental Illness, Pro Persona Mental Health, Wolfheze, The Netherlands.
25 Lentis Research, Lentis Psychiatric Institute, Groningen, The Netherlands.
26 Center for Youth with Psychosis, Mediant ABC Twente, Enschede, The Netherlands.
27 Department of Psychotic Disorders, GGZ-Drenthe, Assen, The Netherlands.
28 Department of clinical and developmental neuropsychology, faculty BSS, University of Groningen, Groningen, The Netherlands.
29 Department ABC Early Psychosis, Altrecht Psychiatric Institute, Utrecht, The Netherlands.
30 GGZ Delfland, Delfland Institute for Mental Health Care, Delft, The Netherlands.
31 Community Mental Health, Mental Health Service Noord-Holland Noord, Schagen, The Netherlands.
32 Early Intervention Psychosis Team, GGZ inGeest Specialized Mental Health Care, Hoofddorp, The Netherlands.
33 Parnassia Psychiatric Institute, The Hague, The Netherlands.
34 GGZ Breburg, Tilburg, The Netherlands.
35 Janssen-Cilag B.V., a Johnson & Johnson company, Breda, The Netherlands.

36 Psychologist Netherlands, Psychosis department, Utrecht, The Netherlands.

37 Behavioural Science Institute, Radboud University, Nijmegen, The Netherlands.

38 Department of Psychiatry, University of Oxford, Oxford, United Kingdom

39 Department of Psychiatry, Amsterdam UMC, Academic Medical Center, Amsterdam, The Netherlands.

40 Department of Pathology, Erasmus MC, Rotterdam, The Netherlands.

41 Rob Giel Onderzoekscentrum (RGOc), Groningen, The Netherlands
